# Do the “big four” orders of insects comprise evolutionarily significant higher taxa with coherent patterns of selection on protein-coding genes?

**DOI:** 10.1093/evlett/qraf005

**Published:** 2025-03-06

**Authors:** Pierre J Février, Timothy G Barraclough

**Affiliations:** Master de Biologie, École Normale Supérieure de Lyon, Université Claude Bernard Lyon 1, Université de Lyon, Lyon Cedex, France; Department of Biology, University of Oxford, Oxford, United Kingdom; Department of Biology, University of Oxford, Oxford, United Kingdom

**Keywords:** higher taxa, protein evolution, conserved, selection

## Abstract

Species are often treated as evolutionarily significant units of diversity that reflect patterns of gene flow and selection. In contrast, higher taxa are mostly regarded as convenient labels for levels in the tree of life, which reflect evolutionary history if defined cladistically but are assumed to have no real significance for ongoing evolution. We test the alternative hypothesis that some higher taxa are evolutionarily significant units with coherent patterns of selection on their constituent species. Specifically, we ask whether the big 4 orders of holometabolous insects, namely Coleoptera, Diptera, Hymenoptera, and Lepidoptera, display divergent, but internally conserved patterns of selection acting on protein-coding genes. Analyzing orthologous genes from whole genome sequence data for multiple species per order, we find that, in most genes, selection on roughly one fifth of codons is conserved within each order but differs significantly among orders. The shift is associated with variation in GC content among orders, but primarily at codon 2nd positions hence due to selection rather than mutational or repair bias. Comparison of alternative models assigning different taxonomic levels (either more lumped or divided than orders) shows that best models always specify Hymenoptera and Lepidoptera as coherent units, whereas patterns of selection on protein-coding genes within Coleoptera and especially Diptera are better explained by subdividing them further. We hypothesise that some aspect of the general lifestyle, body plan or genetic makeup of orders (or of nested clades within Coleoptera and Diptera) leads to conserved patterns of selection across protein-coding genes within them, whereas constraints differ among them. The emergence of whole-genome data for broad and deep phylogenetic samples will allow this hypothesis of evolutionarily significant higher taxa versus more evenly dispersed shifts in selection across genes to be tested further.

## Teaser

Higher taxa are normally considered to be arbitrary levels in the branching tree of life, with no level having greater or lesser significance for evolutionary processes than any other. But are some higher taxa more significant than others for evolution? This article asks whether the big four orders of holometabolous insects comprise evolutionarily significant units showing divergent but internally conserved patterns of selection on protein-coding genes.

## Introduction

Many biologists view species as an evolutionarily significant unit (De Queiroz, 2007) that encompasses both the description of biodiversity and the processes behind its origins (Rieseberg et al., 2006; Wilkins, 2017). Species evolve largely independently because of little or no gene flow among them and because conspecific individuals share cohesive selection pressures (for example, due to their ecological niche) that differ from those faced by other species (Coyne & Orr, 2004). The outcome is a discrete pattern of genetic and phenotypic clusters that can be used to delimit species (Mallet, 1995; Rieseberg et al., 2006), albeit with many challenges in practice due to fuzzy boundaries and a continuum of stages of divergence. Species not only share a common evolutionary history but also a shared evolutionary fate by virtue of shared gene flow and cohesive responses to selection (Barraclough, 2019; Maddison & Whitton, 2023; Templeton, 1989).

In the past, greater importance was given to higher taxa such as genera and families as “natural” units of diversity, from Linnaeus to the mid twentieth century (Simpson, 1953). For instance, a survey in 1940 showed that botanists at the time thought of genera as more ‘natural’ biological units than species (Anderson, 1940; Barraclough & Humphreys, 2015). The emergence of cladistics in the late sixties, however, shifted the focus from potential evolutionary inter-dependence within higher taxa to purely their evolutionary history (Hennig, 1966). Systematics focuses on whether certain groups are monophyletic or not (e.g., Hampl et al., 2009; Kristensen, 1975; Rodríguez-Ezpeleta et al., 2005) and searches for synapomorphies as evidence of monophyly and common ancestry (Assis & Rieppel, 2011). Higher taxa are used for classification, but they are usually not considered to constitute evolutionarily significant units (ESUs), except when used in the fossil record because resolution is too limited to work at the species scale (e.g., De Grave et al., 2009; Miller, 1999; Schachat & Labandeira, 2021; Sepkoski, 1992). Higher taxa provide a convenient shorthand for naming levels within the tree of life (e.g., Shull et al., 2001; Soltis et al., 2007), but no level is deemed to have more biological importance than any other level, except species.

Despite this recent history, it is possible that processes act on larger scales to shape units defined at higher taxonomic levels (Barraclough, 2010). One of these is limits to diversity and turnover due to ongoing speciation and extinction (Barraclough, 2019). If there are constraints on the number of species that co-occur within a geographical region or broadly defined adaptive zone (Simpson, 1953; Stebbins, 1974), and if shifts into that region or zone are relatively rare, then this can lead to the emergence of distinct higher evolutionary significant units that occupy the region or zone (Humphreys & Barraclough, 2014). Species within the zone share both evolutionary history (because of coalescence back to a recent common ancestor at a time defined by the rate of species turnover, i.e., speciation and extinction, within the zone) and evolutionary fate (because the chance of leaving descendant species at a later generation is a zero-sum game among all current species within the zone). Simulation models demonstrated how this can lead to a phylogenetic signature of differentiated clades on relatively long stem branches and statistical methods documented such patterns in mammals, birds and gymnosperms (Humphreys & Barraclough, 2014; Humphreys et al., 2016).

Another scenario that might lend evolutionary significance to higher taxa is if selection pressures on a broad suite of traits and genes are conserved within taxa but divergent among taxa. In general, selection pressures that operate on lineages might be expected to change over evolutionary time at random (Bastide et al., 2018). Closely related species will tend to share more selection pressures than distantly related species and shifts in selection pressures might occur at a constant random rate across the phylogenetic tree, with no concentration of shifts in multiple genes or traits with a particular higher clade or taxonomic level. Alternatively, members of some higher taxa might share a major suite of selection pressures and adaptations, which are divergent from their sister clades. For example, the clade could have invaded a particular adaptive zone or lifestyle that is shared by all its species, such as powered flight in bats (Miller et al., 2023). Humphreys and Barraclough (2014) found patterns of coherence within and divergence among delimited higher taxa for body mass and some other traits in mammals (Euungulata and Carnivora). Species differentiate along additional niche and environmental axes, but there remain conserved features that experience uniform selection pressures within the clade. There could also be a shared body plan or set of genetic pathways that impose constraints on how traits and genes can co-adapt that is distinct from other such clades (Galis et al., 2018).

In this alternative model, understanding how selection has shaped genetic and trait variation requires specification of the clades that experience shared versus divergent selection pressures. Similar ideas underlie comparison of conserved versus novel genetic pathways in evolution and development, where conservatism is used to identify important core pathways and as a backdrop for the emergence of novel traits (Hudry et al., 2014; Saenko et al., 2008). But here the question is where do shifts in selection pressures on genes tend to occur on the phylogenetic tree? Is there a gradual decay in conservatism with increasing phylogenetic distance? Or do shifts occur for suites of genes on the same phylogenetic branches, and if so, do those branches conform to higher taxa traditionally recognized identified in the past for their morphological coherence?

We investigate these questions using whole genome data for representatives of the ‘big four’ insect orders: Diptera, Lepidoptera, Hymenoptera, and Coleoptera. These orders within the Holometabola contain a large fraction of animal species with over a million species altogether. Thus, considered as “megadiverse,” they represent the main sub-radiations in insects and 95% of Holometabola diversity. Holometabolous insects undergo complete metamorphosis and four developmental stages: egg, larva, pupa, and imago. These developmental characteristics are thought to have played a role in the success of the lineage as a whole, but seven additional orders of Holometabola (Peters et al., 2014) are not nearly as diverse as the four major orders, so their mega diversity is usually explained with independent and order-specific phenomena (Grimaldi & Engel, 2005; Hedges & Kumar, 2009). The angiosperm diversification during Cretaceous is associated with the diversification of Lepidoptera (Kawahara et al., 2019), but this link is not as straightforward for other orders. For example, the diversity of Hymenoptera has been linked to parasitism more than the emergence of flowering plants (Beutel et al., 2011), and Coleoptera have diversified across a range of lifestyles, not just those directly associated with plants (Hunt et al., 2007; McKenna et al., 2019). The big four orders have diversified greatly since they split, and phylogenetic relationships within and among orders have been difficult to disentangle (Misof et al., 2014). Yet, the orders themselves have stood the test of time, consistent with a degree of coherence in their characteristics.

**Figure 1.**
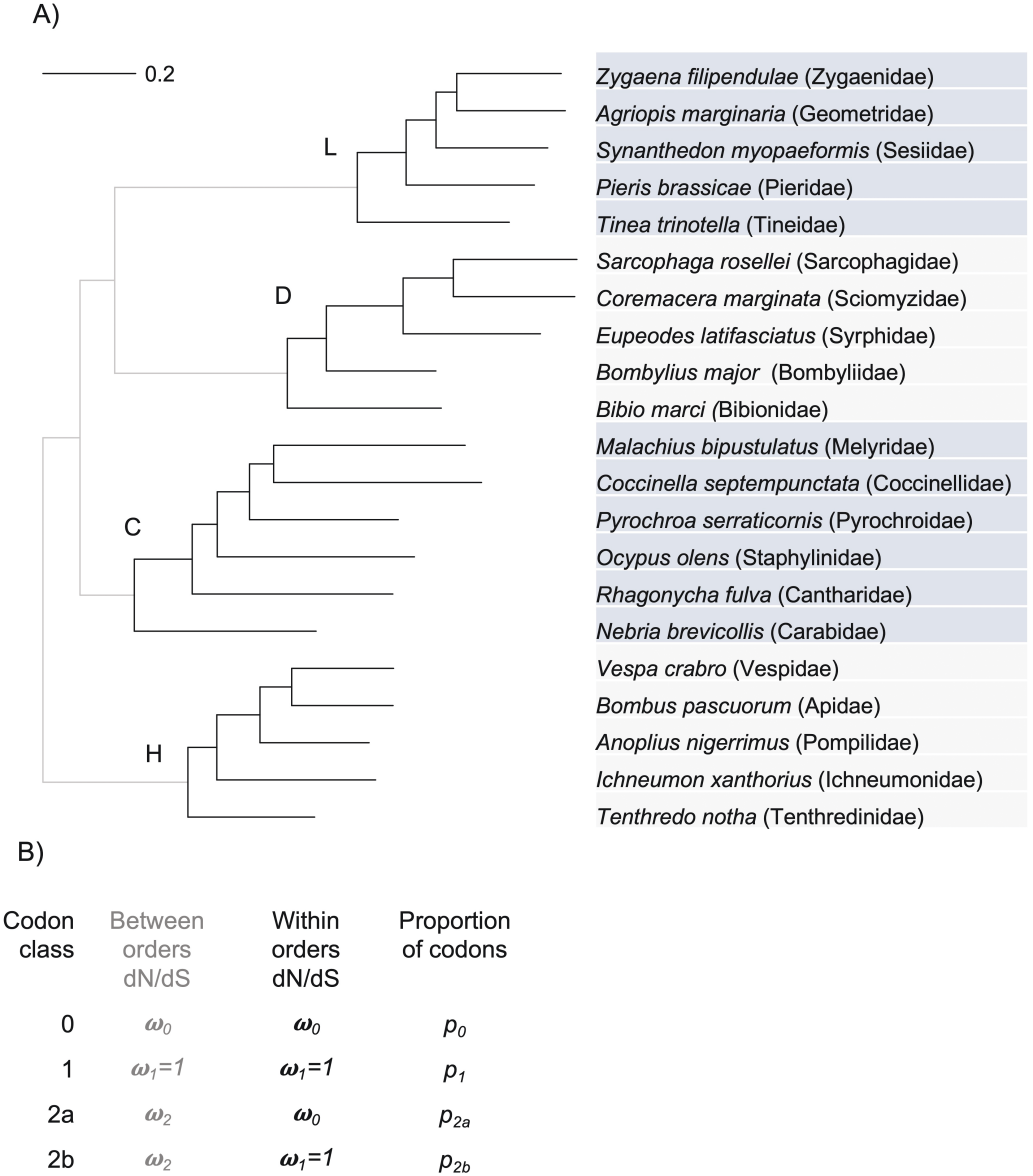
(**A)** Phylogenetic relationships among representative species (and families) of the big four holometabolan orders reconstructed from gene trees of orthogroups: H—Hymenoptera, C—Coleoptera, L—Lepidoptera, D—Diptera. The maximum likelihood tree for a concatenated alignment reconstructed by IQ-TREE2 with the best model chosen using the Bayesian Information Criterion (GTR + F + I + G4) is shown. Scale bar shows branch lengths in units of substitution per site. All nodes have approximate Likelihood Ratio Test (aLRT) support values = 1.0. Within-order branches are in black and among-order branches are in gray. The hypothesis that orders are evolutionarily significant higher taxa predicts a pattern of conserved or purifying selection within orders (i.e., on black branches) but neutral or divergent selection among them (i.e., gray branches), such that purifying selection is structured by orders. (**B)** Summary of the branch-site codon substitution models that were fitted using the PAML software to test the hypothesis.

Here, we test whether insect orders represent evolutionarily significant units, defined as sets of lineages that share conserved selection pressures on their protein-coding genes but are divergent from other such sets (**Figure 1A**). Such a pattern could arise because of conservatism of broad lifestyle strategies within each order, which then differ among them, and from divergent selection on developmental gene networks associated with body plan differences (Peel, 2008). We took advantage of insect genomes emerging from the Darwin Tree of Life Project Consortium (2022) to test alternative models of selection. We aligned groups of orthologous genes (orthogroups) from complete proteomes for a set of species from each of the four major orders. We then fitted different models of selection based on relative rates of nonsynonymous and synonymous substitutions (d*N*/d*S* ratios) across reconstructed gene trees, including models where a subset of codons experience conserved selection within each order, and either unconstrained or positively selected divergence among them (Zhang et al., 2005). Patterns of conservation within orders but divergence among them would be consistent with evolutionarily significant higher taxa that shape and are shaped by patterns of conserved selection. Relatively few genomes were available at the time of our analyses, and so results should be viewed as an initial test of this hypothesis.

## Materials and methods

### Dataset collection

Fully sequenced and annotated genomes were obtained from the Darwin Tree of Life Project website (2023), which is a collaborative project that aims to sequence genomes from 70,000 eukaryotic species in Britain and Ireland to high quality. Among all the fully annotated genomes of Holometabola available on 9th February 2023, five species were selected for Diptera, Lepidoptera and Hymenoptera, and six for Coleoptera (**Table 1**). Species were chosen to represent different families that covered the most phylogenetic diversity possible with the data available at the time. For each organism, FASTA files of unmasked genomes and GFF3 annotation files were downloaded. Coding and protein sequences were extracted using the gffread program (Pertea & Pertea, 2020).

**Table 1.** Species and genome assemblies

Order	Family	Species	Assembly identifier	Genome size Mb	GC %	BUSCO score	Reference
Coleoptera	Carabidae	*Nebria brevicollis*	icNebBrev1.1	242	32.6	98.4	(Crowley & Garner, 2023)
	Coccinellidae	*Coccinella septempunctata*	icCocSept1.1	399	36.4	97.5	(Crowley, 2021b)
	Cantharidae	*Rhagonycha fulva*	icRhaFulv1.1	425	30.4	98.9	(Crowley, 2021a)
	Melyridae	*Malachius bipustulatus*	icMalBipu1.1	544	35.5	98.0	(Crowley, 2021c)
	Pyrochroidae	*Pyrochroa serraticornis*	icPyrSerr1.2	249	34.3	99.5	(Sivell, 2021)
	Staphylinidae	*Ocypus olens*	icOcyOlen1.1	1084	32.6	99.3	(Crowley, 2021d)
Hymenoptera	Pompilidae	*Anoplius nigerrimus*	iyAnoNige1.1	624	45.6	95.0	(Falk & Broad, 2022a)
	Ichneumonidae	*Ichneumon xanthorius*	iyIchXant1.1	315	42.7	95.5	(Broad, 2022)
	Tenthredinidae	*Tenthredo notha*	iyTenNoth1.2	253	37.1	95.5	(Falk & Broad, 2022b)
	Vespidae	*Vespa crabro*	iyVesCrab1.2	230	31.9	96.5	(Crowley, 2022)
	Apidae	*Bombus pascuorum*	iyBomPasc1.1	308	36.5	97.8	(Crowley et al., 2023)
Diptera	Sciomyzidae	*Coremacera marginata*	idCorMarg1.1	980	33.8	97.2	(Sivell & Sivell, 2021a)
	Bibionidae	*Bibio marci*	idBibMarc1.1	340	25.6	91.8	(Sivell & Sivell, 2021b)
	Sarcophagidae	*Sarcophaga rosellei*	idSarRose1.1	541	33.4	99.0	(Falk & Mulley, 2023)
	Syrphidae	*Eupeodes latifasciatus*	idEupLati1.1	846	33.5	96.3	(Falk & Chua, 2022)
	Bombyliidae	*Bombylius major*	idBomMajo1.1	304	25.9	94.8	(Lawniczak et al., 2023)
Lepidoptera	Tineidae	*Tinea trinotella*	ilTinTrin1.1	372	35.7	94.6	(Boyes & Chua, 2022)
	Pieridae	*Pieris brassicae*	ilPieBrab1.1	292	33.8	99.0	(Lohse & Mackintosh, 2021)
	Geometridae	*Agriopis marginaria*	ilAgrAura1.1	501	38.1	98.3	(Lees & Boyes, 2023)
	Sesiidae	*Synanthedon myopaeformis*	ilSynMyop1.1	296	33.9	98.0	(Langdon & Holland, 2024)
	Zygaenidae	*Zygaena filipendulae*	ilZygFili1.2	366	36.6	97.8	(Boyes et al., 2022)

### Identification, alignment, and tree reconstruction of orthologous genes (orthogroups)

Orthologous genes were inferred across the whole dataset using OrthoFinder v2.5.4 (Emms & Kelly, 2019). The input files were amino acid sequences for each gene in each genome. Following assignment to orthogroups (23,644 in total), amino acid sequences of each orthogroup were back-translated to nucleotides sequences with the aa_to_dna_aln utility from the Bio::Align::Utilities BioPerl module (and nucleotide transcript fasta files) then realigned using the DECIPHER package from the Bioconductor (v3.17) project in R (Wright, 2016). This provided nucleotide alignments needed to model d*N*/d*S* ratios. Orthogroups with between 10 and 100 sequences and at least 1 sequence belonging to each order were carried forwards (5,219 in total) to avoid working with too few samples across species or with gene families that were too large. Identical sequences and those with over 66% of gaps were discarded, then any columns of alignment with codon gaps were also deleted to produce gap-free alignments suitable for input to later steps (with scripts in R using the ape package, Paradis et al., 2004). Gene trees were reconstructed for each orthogroup by maximum likelihood and a GTR + I + G substitution model in IQ-TREE2 (Nguyen et al., 2015).

One complication for our analyses was the presence of additional copies of genes (paralogs) within a given orthogroup. To minimize the confounding effect of functional divergence between paralogs on later analyses, we performed the following filtering steps (R scripts available at https://github.com/tim-barra/Fevrier2024). First, some orthogroups displayed evidence of ancient duplication such that multiple clades are present that each contain members of all four orders (**Supplementary Figure S1**). For each orthogroup, we therefore extracted the most nested sub-clade in the tree containing all four orders and deleted all other sequences from the alignment (**Supplementary Figure S1**). Second, in some orthogroups, one or more species contained two or more copies of the gene consistent with recent duplication. For all orthogroups, we therefore randomly deleted any duplicate copies within species to retain only a single copy per species (after completing filtering step 1, **Supplementary Figure S1**). Third, in some cases a single highly divergent sequence is evident on the tree even after the above steps. We therefore discarded the top 5% of orthogroups after steps 1 and 2 with respect to maximum branch length in their maximum likelihood tree (indicative of highly divergent sequences). Removing any filtered alignments that had fewer than 10 sequences after these steps left a total of 4,851 orthogroups (sum of alignment lengths = 3,231,261 base pairs).

We used two different datasets for subsequent analyses. First, we constructed a concatenated alignment. Because the software used to fit substitution models assumes no missing data, we concatenated the subset of orthogroups (*N* = 2,034, alignment length 1,525,512 base pairs) that included a sequence for all 21 species in our sample. Second, we ran analyses separately on each orthogroup in turn, which allowed us to consider the full set including orthogroups missing one or more species.

### Codon models of selection

We used the ratio of nonsynonymous-to-synonymous substitutions to test the mode of evolution, defined as *ω = *d*N*/d*S*. Synonymous mutations are silent and presumed neutral, while nonsynonymous mutations change an amino acid and could be under selection. A ratio>1 indicates that positive selection is driving a faster rate of amino acid change than the expected rate of neutral changes, a ratio of 1 is consistent with neutral evolution and a ratio <1 indicates purifying selection against amino acid changes (Jeffares et al., 2015; Kryazhimskiy & Plotkin, 2008). We implemented four main models for each orthogroup in turn using the CODEML program in PAML v4.10.6 (Yang, 2007).

*A nearly neutral site model* (called 1a in PAML, here shortened to model 1) estimates mean d*N*/d*S* ratios and the proportions (*p*_*0*_ and 1 − *p*_*0*_) of codons belonging to each of two classes. Class 0 codons are under purifying selection (*ω*_*0*_ < 1) and class 1 codons are neutral (*ω*_*1*_ = 1). It assumes that class 0 codons are constrained to encode the same amino acid across the whole tree.

*A positive selection site model* (called 2a in PAML, here shortened to model 2) estimates mean d*N*/d*S* ratios and the proportions of codons belonging to each of three classes, purifying (*p*_*0*_, *ω*_*0*_ < 1), neutral (*p*_*1*_, *ω*_*1*_ = 1), and adaptive (1 − *p*_*0*_- *p*_1_, *ω*_2_* *> 1). It assumes that amino acid changes in class 2 codons under positive selection are distributed across the whole tree, rather than localized on particular branches.

*A nearly neutral branch-site model* (which we call model 3 here) assumes that codons are either under purifying selection or neutral as in the nearly neutral model 1, but a class of codons that are constrained on background branches (*ω*_0_ < 1, within orders, gray branches, **Figure 1**) display a pattern of “neutral” evolution on (*ω*_2_ fixed to 1) foreground branches connecting different orders (black branches, **Figure 1**). This codon class is constrained within orders but unconstrained in their amino acid divergence among orders.

*A positive selection branch-site model* (we call model 4 here) assumes the same three classes of codons as the positive selection site model 2 but that a class of codons (called 2a in PAML (Yang, 2007)) are constrained on background branches (*ω*_*0*_ < 1, within orders, gray branches, **Figure 1**) but experience positive selection (*ω*_2_ > 1) on foreground branches connecting different orders (black branches, **Figure 1**). Another class of codons (2b in PAML) shift from neutral within orders (*ω*_1_ = 1) to potentially positive (*ω*_2_ > 1) among orders.

Accepting either model 1 or 2 would reject the importance of insect orders for patterns of selection on protein-coding genes, because the same average substitution model is fitted within and among them. Both models assume amino acid changes are randomly or evenly distributed across the whole tree. In contrast, accepting either model 3 or 4 would support the existence of a category of codons that are constrained by purifying selection within orders but either unconstrained (model 3) or under positively selected divergence (model 4) among them.

### Model comparison

We fitted all four models to the concatenated alignment and each separate orthogroup alignment in turn and retrieved the likelihood score for each model. We assumed the F3X4 codons substitution model but repeated a subset of analyses with the empirical codon frequencies model to check whether it affected conclusions. PAML adopts a frugal approach to specifying models in the branch-site model, by placing constraints on the proportions of codons belonging to different categories and allowing only three d*N*/d*S* ratios (one of them fixed at 1). Branch-site model 4 consequently has the same number of free parameters as the site model 2, namely 4, despite containing additional structure (the subset of codons that switch categories between foreground and background branches). Nested log likelihood ratio tests therefore cannot be used to compare them. Instead, we used the Akaike Information Criterion (AIC = −2 × log likelihood + 2 × number of parameters) to select the best model for each orthogroup and AIC weights to calculate the relative likelihood of the four models. AIC weights were calculated as exp(−0.5 × deltaAIC), where deltaAIC = AIC—AICmin, and AICmin is the minimum AIC score across the four models for each orthogroup (Burnham & Anderson, 2004). We checked for uninformative parameters between the two site models and two branch site models in turn (ie, if *ω*_*2*_ is estimated as 1.0, the same as *ω*_*1*_). In such cases, we excluded models 2 and 4 with uninformative parameters prior to AIC comparison as recommended by Leroux (2019). Further analyses explored possible effects of saturation of synonymous substitutions on these comparisons (Supplementary Methods).

### Analysis of codon positions and the effects of GC content on substitution patterns

To check results of model fitting in PAML, we visualized codon substitution patterns across the tree using an alternative method. We fitted branch lengths to the maximum likelihood tree for the whole concatenated alignment for (a) just 2nd position sites (largely nonsynonymous) and (b) just 3rd position sites (largely synonymous), in turn, using a GTR model in IQ-TREE2. We then calculated the ratio of 2nd position branch lengths to 3rd position branch lengths as a surrogate metric of the d*N*/d*S* ratio on each branch and plotted these values on the tree. We used ANOVA to compare the average ratio of 2nd to 3rd position sites on among-order branches versus within-order branches, as an alternative test of the “order” effect. (We were unable to estimate d*N* and d*S* separately on each branch in PAML due to computational limitations of the “one-per-branch” model).

GC content has been reported to be higher in coding regions of Lepidoptera than the other three big orders of Holometabola, which is argued to result from GC-biased gene conversion. In principle, this could influence patterns of protein divergence (as reported in mammals, Dai et al., 2020; Hargreaves et al., 2017), or amino acid divergence might be unaffected (if GC bias is observed primarily in silent substitutions, as reported in a previous insect study, Kyriacou et al., 2024). We therefore checked for GC bias among our sampled genomes by testing for variation in the GC content of 1st, 2nd and 3rd position sites among orders using ANOVA. Across the separate orthogroups, we tested whether the proportion of codons in the class 2a that shift among orders covaries with high variance in mean GC content among orders.

### Do orders reflect the best taxonomic grouping to structure patterns of gene evolution?

We chose “orders” as a prior hypothesis for higher taxa that might shape patterns of protein-coding evolution based on their importance for describing insect diversity. But in principle, other taxonomic levels might represent a better structure to define patterns of protein evolution, for example splitting orders into sub-lineages or lumping orders into even higher taxonomic units. To investigate whether “orders” represent the best units for structuring selective constraints on protein coding genes, we ran further versions of the branch-site model. Specifically, for the concatenated matrix, we ran 17 alternative versions of the best branch-site model with different branch labeling each time (shown in **Supplementary Figure S2**): either lumping together orders (2 models—one lumping the two most derived orders together, one lumping the three derived orders together into a single unit), or dividing each order at its basal node: either one at a time (4 models), two at a time (6 models), three at a time (4 models), or all four orders subdivided (1 model). We then used the Akaike Information Criterion (AIC) weights of these different structure models (all with the same number of parameters), and of the original model with orders as units, to see which model had strongest support.

## Results

### Around 20% of codons are under strong purifying selection within orders but divergent among orders

Four substitution models were fitted to the concatenated alignment of the 2034 orthogroups having a sequence for all 21 sampled species (assuming the F3X4 codon substitution model): two site models, the nearly neutral (model 1) and positive selection (model 2); and two branch-site models, a nearly neutral version (model 3) and positive selection version (model 4). The extra parameter in model 2 was uninformative compared to model 1 and the extra parameter in model 4 was similarly uninformative relative to model 3 (in both cases estimated as *ω*_*2*_ = 1.0, that is, indistinguishable from *ω*_*1*_, **Table 2**) and so models 2 and 4 were excluded from model comparison (Leroux, 2019). There was strong support for model 3 (logL = -22411539, 3 free parameters, AIC weight = 1.00) over model 1 (logL = -22475410, 2 free parameters, deltaAIC = 127740.4, weight = 0.00, **Table 2**). An estimated 73.0% of codons were under purifying selection across the whole tree (class 0 codons, *ω*_*0*_ = 0.04), 5.6% evolved neutrally across the whole tree (class 1 and 2b codons combined in the constrained model, *ω*_*1*_ = 1), whereas 21.4% (class 2a codons) were under purifying selection within orders (*ω*_*0*_ = 0.04) but showed unconstrained divergence among them (*ω*_*2*_ = 1.0). Conclusions were the same using the empirical frequency codon model instead of the F3X4 model (same AIC weights for model 1 and model 3, 23.9% of codons are in set 2a, **Table 2**). The reconstructed tree for the concatenated matrix under model 1 had a maximum patristic pairwise dS between tips of 8.03 substitutions per codon, and conclusions were robust to potential effects of saturation of synonymous substitutions (Supplementary Text, **Table S1**, **S2**, **Figure S3**).

**Table 2.** Codon substitution model results for the concatenated matrix.

Dataset	Codon	Model			Proportion in each class				d*N*/d*S* ratios			Log lik	AIC	AICw
	Model	#	Type	Nsite	*p*_*0*_	*p*_*1*_	*p*_*2a*_	*p*_*2b*_	*ω*_*0*_	*ω*_*1*_	*ω*_*2*_			
Full	F3 × 4	1	S^*^	NN^*^	0.940	0.060			0.041	1		−22475410	44950824	0.00
Full	F3 × 4	2	S	P	0.940	0.035	0.024		0.041	1	1	−22475410		
Full	F3 × 4	3	BS	NN	0.730	0.043	0.214	0.013	0.040	1	1	−22411539	44823084	1.00
Full	F3 × 4	4	BS	P	0.730	0.043	0.214	0.013	0.040	1	1	−22411539		
Full	Fcodon^*^	1	S	NN	0.944	0.060			0.035	1		−22370347	44740698	0.00
Full	Fcodon	3	BS	NN	0.708	0.040	0.239	0.013	0.035	1	1	−22309070	44618146	1.00

^*^Fcodon refers to the empirical codon frequency model, S = site, BS = branch-site, NN = Nearly neutral, P = positive selection. Additional parameters as defined in the text.

**Figure 2.**
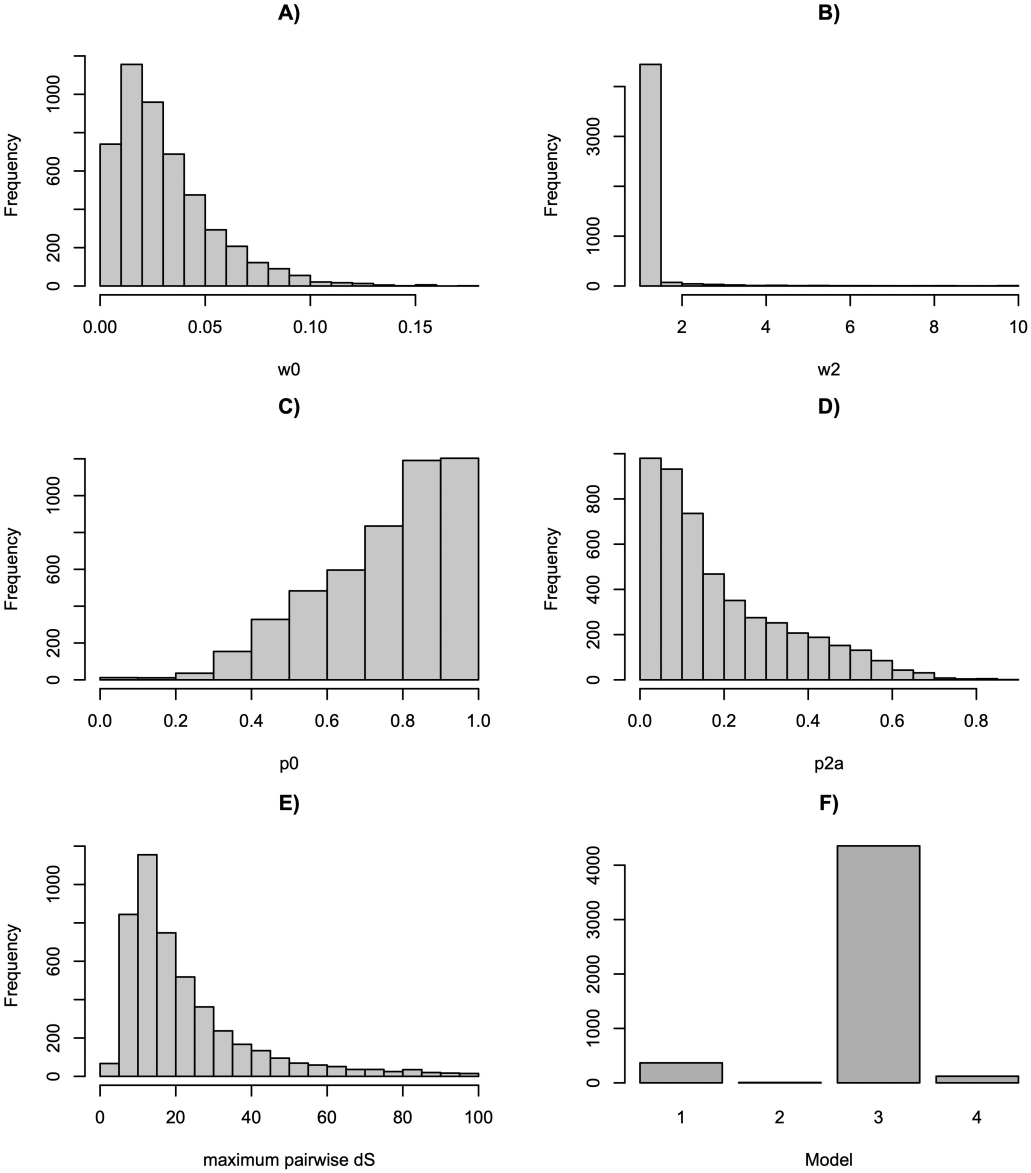
The distribution of parameter estimates and other metrics across the separate analyses of 4,851 orthogroups. Values estimated under model 4 (positive selection branch-site model) are shown. (**A)** The dN/dS ratio of codons under purifying selection. (**B)** The estimated d*N*/d*S* ratio of codons shifting to neutral or positive selection on among-order branches. (**C)** The proportion of codons in the class that are under purifying selection across the tree. (**D)** The proportion of codons shifting from purifying selection within-orders to either unconstrained (neutral) or positive selection among-orders. (**E)** The maximum pairwise dS (number of synonymous substitutions per codon) per orthogroup. (**F)** The number of orthogroups supporting each model: 1 (nearly neutral site), 2 (positive selection site), 3 (nearly neutral branch site) and 4 (positive selection branch-site).

Separate analysis of 4,851 orthogroups containing at least one sample from each order (on average 4.7 sequences were included per order, **Table S3**) also provided wide support for Model 3 (**Figure 2**), which had the lowest AIC of the four models in 4,354 (89.8%) of orthogroups (**Figure 2F**, example gene tree and alignment in **Figure S4**, model outputs in **Table S4**). On average, 18.7% of codons were estimated to experience a shift from strong purifying selection within orders to unconstrained among orders to (50% of estimates across orthogroups fall between 6.3% and 27.6%, **Figure 2D**). Conclusions were robust to variation in parameter estimates due to saturation of synonymous substitutions (Supplementary Text, **Table S5**). Model 1 was preferred in 367 (7.6%) orthogroups, model 2 in 7 (0.1%). Only 122 (2.8%) of orthogroups had the lowest AIC for model 4, namely a shift to positive selection on among-order branches (for these cases, mean *ω*_*2*_ = 6.2, with a long skew, median *ω*_*2*_ = 1.2, **Figure 2B**; mean proportion of codons in class 2a for these orthogroups = 24.2%).

### GC content varies among orders in a pattern indicative of selection

**Figure 3.**
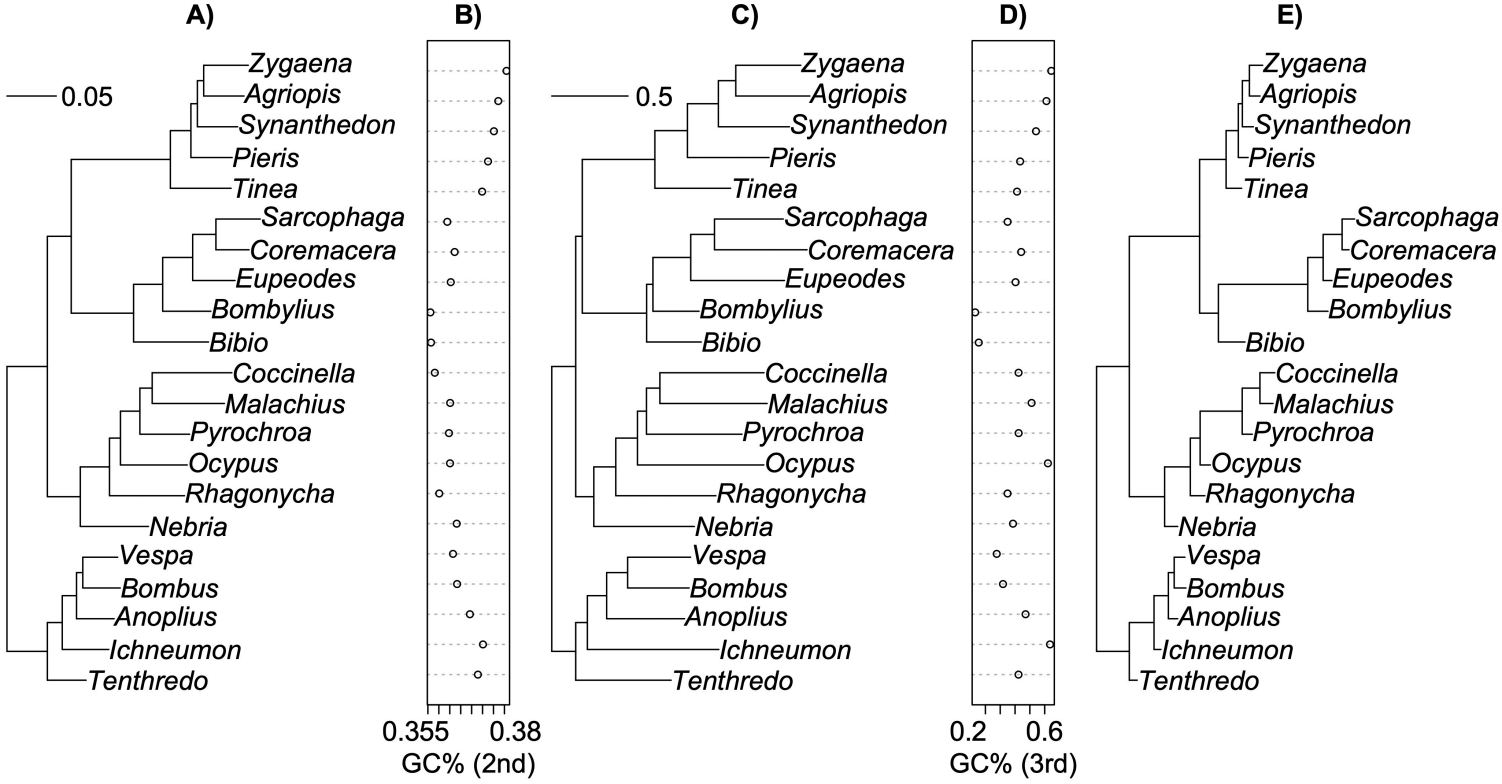
The maximum likelihood tree for the concatenated alignment with branch lengths reconstructed for (**A)** just 2nd position sites, (**C)** just 3rd position sites, and (**E)** with branch lengths depicting the ratio of 2nd to 3rd position branch lengths. Average GC content at (**B)** 2nd position sites and (**D)** 3rd position sites is also shown, note the different scale for each plot as 2nd position GC content varies over a narrower range than at 3rd positions, but with significant differences among orders.

We explored variation at 2nd and 3rd position sites of codons to investigate model results further (**Figure 3**). The ratios of branch lengths reconstructed for 2^nd^ and 3^rd^ position sites displayed the same pattern as PAML model comparisons (**Figure 3E**): among-order branches display a significantly higher ratio of 2nd:3rd position substitutions than within-order branches (mean ratio among = 0.29, within = 0.13, *F*_1,38_ = 8.7, *p* = .0053), consistent with higher rates of d*N* relative to d*S* changes on among-order branches. A few cases of short 3rd position branches are apparent, associated with a high 2nd:3rd ratio, but such cases are observed both within (eg, the branch leading to *Pyrocrhoa*, *Malachius,* and *Coccinella* within beetles) and among (eg, the branch leading to Diptera plus Lepidoptera) orders.

GC richness across all sites varies significantly among orders in the whole concatenated alignment (*R*^2^ = 0.27, *F*_3,17_ = 3.48, *P* = .039, Supplementary Figure S5A). Lepidoptera has the highest GC% on average (48.3 + 1.9%) and Diptera lowest (38.2 + -2.7%). The pattern differs across codon positions, however. GC richness varies widely at 3rd positions (13.1% in *Bombylius* to 64.4% in *Zygaena*, **Figures 3D,****Supplementary Figure S5A**), but marginally insignificantly among orders (*R*^2^ = 0.24, *F*_3,17_ = 3.16, *P* = .052), because of wide variation within each order. In contrast, GC content varies little at 2nd positions across the entire sample (35.6% in *Bombylius* to 39.1% in *Zygaena,***Figure 3B,****Supplementary Figure S5A**), but nonetheless highly significantly among orders (*R*^2^ = 0.78, *F*_3,17_ = 25.8, *P* < .0001). GC content variation therefore appears to contribute to amino acid divergence among orders, but shifts in mutational or gene conversion bias in GC among orders do not seem to drive that pattern: if they did, we would expect the strongest effect of “order” on GC content at 3rd position sites. Variation in amino acid composition among orders covaries with whether the 2nd position is G/C or A/T, for example, being stronger for amino acids determined by G or C at two positions compared to just one position (Supplementary Figure S6).

As a further test, we examined codons identified as having a high probability (*P* > 0.95) of belonging to the 2a class from the Bayes Empirical Bayes (BEB) sampling output from model 4 applied to the concatenated alignment (*n* = 36489 codons, 7% of whole alignment). Across these codon positions, GC% at 2nd position sites show an even stronger pattern of significant variation among orders (*R*^2^ = 0.94, *F*_3,17_ = 105.8, *P* < .0001, **Supplementary Figure S5B**) as across all codons. The global average GC% for these BEB identified codons across all orders is also higher, being 47.6 + 0.2% compared with 37.1 + -0.8% across all codons.

### Model fits improve when splitting Coleoptera and especially Diptera

To test whether insect orders are the best structure to explain limits of purifying selection, we compared the AIC scores of seventeen alternative structures for model 3 that either lumped orders together or split each order (either alone or in all combinations) at its basal node, running for the whole concatenated alignment (**Supplementary Figure S7**, model results in **Supplementary Table S6**). The best supported models in decreasing order of support were: splitting both Coleoptera and Diptera (CD), just splitting Diptera (D), just splitting Coleoptera (C) and the original model of orders. Combined AIC weight for model CD was >99.9% confidence. Models that lumped orders together were always much worse than the original model of orders, as were models that split either Hymenoptera or Lepidoptera (all delta AIC > 28889 log likelihood units, **Supplementary Table S6**). We conclude that Hymenoptera and Lepidoptera constitute higher taxonomic units that define coherent patterns of selection on protein-coding genes, whereas Coleoptera and Diptera contain sub-clades that show separate patterns of purifying selection across the sampled genes and are less coherent in terms of dN/dS variation. Hymenoptera and Lepidoptera similarly comprise more tightly defined clusters than Coleoptera and Diptera based on visualizing pairwise amino acid divergence across the full concatenated alignment (**Supplementary Figure S8**).

## Discussion

Our analyses of a sample of holometabolan insect genomes found evidence consistent with higher evolutionarily significant units that shape patterns of selection on protein-coding genes. Although most codons (71%) displayed uniform purifying selection across all taxa, another large set (23%) displayed low d*N*/d*S* ratios within orders (mean = 0.04) but high d*N*/d*S* ratios (≥1) among them. Both Hymenoptera and Lepidoptera were supported as coherent taxa, whereas Coleoptera and Diptera were better split into constituent sub-clades to predict patterns of variation. The results comprised a general feature of the sampled genes. It would be natural to imagine that the phylogenetic location of any shifts in d*N*/d*S* ratio would be randomly distributed for different genes, but that was not the case. For example, the best fit model (CD) of splitting Coleoptera and Diptera was supported by separate analyses on 13% orthogroups whereas only 0.4% supported the opposite model (HL) with Coleoptera and Diptera coherent and Lepidoptera and Hymenoptera both split.

Practical limitations meant that we could not apply our methods to all sequences in all orthogroups identified from the proteomes of each species, and so our filtering steps might have introduced a bias that created a tendency to observe divergence among orders. Orthogroups were mainly rejected, however, due to one or more of the orders being completely missing, which prevented our core model being applied. Across the separate analyses of orthogroups, we found a significantly higher proportion of codons belonging to class 2a that shift among orders when all four orders were monophyletic in the gene tree. Again, this seems like a natural result rather than bias, as it is harder to envisage coherence at a suite of codon sites within orders if the sequences do not group together in the tree: the same branch-site model was still preferred among orthogroups that lacked order-monophyly.

Saturation posed another potential issue with our analyses, as our branches of interest are de facto deeper in the tree and hence might face underestimation of synonymous changes relative to nonsynonymous changes. We found that saturation did affect parameter estimation under model 3, with a tendency to increase the proportion of codons assigned to the class that switch from constrained to unconstrained on among-order branches (Supplementary Text). Yet, the strong preference for model 3 over model 1 seemed not to be explainable by observed levels of saturation in our dataset: only much higher levels of saturation would cause this effect. Overall, our dataset falls within levels of divergence indicated by prior simulations not to greatly affect performance of the models: for example, Yang 1998 FAQ states saturation a problem with over 10 to 50 substitutions per site along the tree, = 30 to 150 per codon, and Gharib & Robinson-Rechavi, 2013 found false positives arise for tests of positive selection only with dS ~ 50 or more.

We focused on orders as the taxonomic rank to test for hypothetical higher evolutionarily significant units because of the available sample of genomes and the importance of orders in describing major different branches of the holometabola. Hymenoptera and Lepidoptera were supported as coherent units whereas Coleoptera and especially Diptera gave better results when split into two sub-clades. Sampling of more taxa would be needed to investigate the best fit limits for purifying selection within each order, and potentially more complex models fitted that have one set of codons constrained at the level of orders whereas another set is constrained at a more resolved level. For example, insect families might represent relevant units to predict ecological properties (Srivathsan et al., 2023), and have been described as natural units that conform to many common names for kinds of insects used in English (unlike tribes, for example, (Barnard, 2011). Future models could also retain paralogous gene copies within analyses, and fit more complex structure models including among-taxon and between-paralog components of functional divergence.

Even if entities that match our concept of discrete units for purifying selection exist, they need not of course correspond to orders or any other named level in the taxonomic hierarchy. However, orders were originally recognized based on natural coherence and divergence in their morphology and lifestyles, which here is demonstrated to be reflected in one aspect of distinctiveness in sequence variation across a sample of protein-coding genes. Humphreys and Barraclough (2014) drew similar conclusions when inferring higher taxa in mammals from patterns of phylogenetic branching: most of the higher ESU corresponded to traditionally named taxa.

What biological processes might generate the pattern reported here? We did not find strong evidence for divergent selection among orders, which was to be expected because tests for positive selection are well known to lack power for deeper branches (Gharib & Robinson-Rechavi, 2013). The finding of strong purifying selection acting within each order (with d*N*/d*S* ratio = 0.04) coupled with a unconstrained, neutral divergence among them is nonetheless indicative of a shift in selective optimum at those codons among orders, otherwise we would expect to observe d*N*/d*S* ratio << 1 on all branches.

It seems unlikely that specific adaptations in each order are responsible, such as the elytra of beetles (Linz et al., 2023) or halteres of flies (Yarger & Fox, 2016), as such traits seem unlikely to impact such a large arbitrary set of protein-coding genes. Key gene regulatory factors can be associated with important developmental and adaptive changes, as is the case for the holometabolous life cycle as a whole (Truman & Riddiford, 2019). The sampled orthogroups included matches to regulatory genes (*n* = 1,573) and those involved in developmental processes (*n* = 1,262). These included several narrower GO functional terms such as “compound eye morphogenesis,” “imaginal disc-derived wing morphogenesis,” “wing vein morphogenesis,” and ‘chitin-based cuticle development’ of potential relevance for holometabolan differences (**Supplementary Table S7**). Yet, the sampled orthogroups displayed matches to a wide range of biological functions, and there was no enrichment for developmental or insect-specific functions in those with the strongest signatures of divergence among orders. Nonetheless, large-scale changes in morphology and lifestyle might entail changes in large numbers of coding sequences, which co-adapt during major transitions.

One clue comes from the analysis of GC content, which showed that orders differ significantly, but primarily at 2nd position sites associated with amino acid changes. This is consistent with selection for amino acids with more GC rich 2nd position sites in some orders (e.g., Lepidoptera, with the highest GC content) than others (e.g., Diptera or Coleoptera). Although the absolute change in GC content is subtle, ranging only from 44.7% in Coleoptera to 52.4% in Lepidoptera even at the subset of codons assigned to class 2a, it is pervasive across a wide sample of genes. What might cause a shift in GC content focused especially on 2nd position sites? We speculated whether this could be an indirect effect of selection on increased or decreased use of essential amino acids (which covaries somewhat with GC, e.g., all amino acids with T at 2nd position are essential in insects, Tomberlin et al., 2023). However, essentiality was less predictive of patterns than GC content of 2nd positions per se (**Supplementary Figure S6**). The mechanism for GC variation at 2nd position sites therefore remains unclear. Rates of genome structure evolution have also been found to vary consistently among orders, with for example Lepidoptera and Diptera showing much higher rates than other orders (Alfieri et al., 2023), indicative of additional major genome changes associated with divergence among orders, with which perhaps GC shifts covary.

Our analyses relied on an extremely small sample of species per order, and omitted the other holometabolan orders, so at this stage should be regarded as raising a hypothesis rather than providing the final answer. Yet, we present initial evidence that orders (or narrower subclades in two cases) constitute evolutionarily significant units in the phylogenetic hierarchy for insect molecular evolution, which can be observed across a broad sample of protein-coding genes. As more taxa become available for whole genome analysis, plus refined methods that account for multiple shifts and paralog divergence, it will be possible to test these ideas further and describe how patterns of selection vary across phylogenomic scales during major transitions.

## Supplementary material

Supplementary material is available online at *Evolution Letters*.

qraf005_suppl_Supplementary_Figures_S1-S8

qraf005_suppl_Supplementary_Tables_S1-S7

## Data Availability

R scripts and concatenated and separate filtered orthogroup alignments and tree files are available at https://github.com/tim-barra/Fevrier2024
