# Supplementary material for: Do the “big four” orders of insects comprise evolutionarily significant higher taxa with coherent patterns of selection on protein-coding genes?
Source: Evol Lett. 2025 Mar 6;9(3):355–66. doi: 10.1093/evlett/qraf005 (PMC12137047; doi:10.1093/evlett/qraf005)

SUPPLEMENTARY TEXT – Fevrier & Barraclough 2025

METHODS

Investigating the potential effects of saturation on model comparisons

Among-order branches are older branches on the tree, and therefore might be expected to exhibit saturation especially for synonymous substitutions. Potentially this could lead to a bias for over-estimating dN/dS ratios on those branches (because dS is artefactually low) and towards accepting a branch-site model (model 3 or 4). To evaluate this potential bias, we performed several further analyses. First, we recorded metrics of dS for each orthogroup and used linear models to test whether PAML model results (specifically the proportion of codons attributed to class 2a that shift among orders) correlate with dS across orthogroups in the separate analysis. We included additional covariates: whether orders were recovered as monophyletic in the orthogroup gene tree or not, variance in mean GC content among orders and the number and length of sequences in the alignment. The model was simplified using the step function in R to obtain the minimum adequate model, and effect sizes reported using partial R2 (the reduction in R2 when removing each term in turn from the model) and the magnitude of fitted changes in the response.

Second, we repeated the concatenated analysis but including only orthogroups within the lower 20th percentile of estimates for maximum pairwise dS (N = 384 orthogroups), to check whether the same pattern is found in the least saturated orthogroups. We also simulated alignments under the best-fitting site model (i.e. with no effect of orders) for the "lower 20%" concatenated alignment using evolveNSsites in PAML, and repeated all analysis steps to compare substitution models. If the model tends to fit lower dN/dS on deeper branches as an artefact of saturation fitting, we might expect to see a bias towards accepting a branch-site model despite data being simulated under a site model. One complication is that simulations use branch lengths estimated under the site model, which might already underestimate lengths of deeper branches and under-represent the true degree of saturation. We therefore repeated the simulations but scaling the among-order branches on the tree used to generate simulated data to be artificially 1.5X, 2X or 5X longer than in the reconstructed tree from the best site model (following the approach of Gharib and Robinson-Rechavi 2013). We generated simulated alignments based on the scaled trees and the best fit site-model for the empirical data, and then ran all steps of tree-building and PAML analyses on the simulated alignments. Thus, by fitting models to these manipulated cases, we tested whether underestimation of synonymous substitution rate due to saturation could lead to artifactual rejection of a site model.

RESULTS

Parameter estimation is affected by levels of saturation but model selection is robust

To investigate whether our model comparisons were affected by saturation of synonymous substitutions on among-order branches, we first fitted linear models to results from the separate analyses of orthogroup alignments. The proportion of codons assigned to class 2a under model 3, which are the codon set that shift from constrained to unconstrained on among-order branches, does increase with log(dS) across orthogroups (partial R2=0.035, **table S5**): on average p2a is 0.13+-0.003S.E. among orthogroups with the lower 20% of dS values, rising to 0.23+-0.007S.E. among orthogroups with the higher 20% of dS values. Model 3 remained strongly preferred, however, among orthogroups within the lower 20% of dS values (853 out of 970 orthogroups, 88%). More of the variation in p2a was explained by whether orders were recovered as monophyletic or not in the gene tree (partial R2= 0.1083, **table S5**): the estimate of p2a doubled when orders were monophyletic (0.24) versus not (0.13). The number of sequences in the orthogroup had a small positive effect on p2a as well (**table S5**), but length of sequences and variance in mean GC content among orders had no significant effect and were removed from the minimum adequate model.

As a second evaluation of the effects of saturation, we re-ran analyses on a concatenated alignment including only orthogroups with the lower 20% of values of the maximum patristic dS distance between any pair of sequences (as estimated fitting model 1 to each orthogroup in the separate analyses). The maximum patristic dS distance estimated for the reduced alignment was 4.81 compared to 8.03 substitutions per site for the entire concatenated alignment. Models 2 and 4 again included uninformative parameters (ω_2=1.0) but there remained strong support for model 3 (logL = - 4356273, 3 free parameters, AIC weight = 1.00) over model 1 (logL = - 4367711, 2 free parameters, deltaAIC= 22873.6, weight = 0.00, **table S1**). Parameter estimates showed a smaller proportion of codons displaying the shift in mode among orders than for the full alignment: 78.0% of codons were under purifying selection across the whole tree (ω_0=0.049), 9.2% evolved neutrally across the whole tree, and 12.8% were under purifying selection within orders (ω_0=0.046) but unconstrained among them (ω_2=1.0).

Our third test for effects of saturation simulated datasets assuming the best site model (model 1, with no effect of orders on substitution patterns) with empirically fitted parameters and a tree with either (a) empirically reconstructed branch lengths (**figure S3A,E**), or (b) among-order branches scaled by 1.5X, 2X or 5X compared to their reconstructed lengths

(**figure S3B,C,D,F,G,H**), in turn. If saturation caused a bias towards accepting model 3 (the branch-site model), we would expect an increasing rate of false acceptance of model 3 as artificially scaled branch lengths increase. In most simulations, including those using the original empirically reconstructed branch lengths, model 3 was indistinguishable from model 1 (i.e. having the same likelihood score and p2a estimated as 0, **table S1**), as expected if model comparison is unbiased for data simulated under model 1 (**table S2**). Only when among-order branches were scaled unrealistically to 5 times their empirically reconstructed length, did model 3 estimate a high proportion of codons (0.84) in class 2a (**figure S3H**), consistent with a potential bias. In this scenario, there were two solutions, one with long among-order branches (as fitted by model 1, **fig S3D**) and one with shorter among-order branches but a higher average dN/dS ratio on those branches due to class 2a codons (as fitted by model 3, **fig S3H**). Yet, model 1 still received AIC weight 1.00 in model comparison with model 3, hence was preferred even in this case (**table S2**). We found no bias therefore towards false acceptance of model 3, even with levels of saturation beyond those estimated for our data.

SUPPLEMENTARY FIGURES

Figure S1. Illustration of filtering steps to remove paralogous gene copies from the final orthogroup alignments. **A)** The unfiltered maximum likelihood gene tree for orthogroup OG0000181. Multiple sequences are present per species, including one highly divergent sequence. We extracted the most nested clade within the gene tree (curved bracket) and then deleted additional copies per species at random (asterisks and strike through) to generate **B)** a filtered alignment and gene tree for the orthogroup with just a single sequence included for each species that is present in that orthogroup. In this example, only *Malachius* is missing from the orthogroup. Note that Coleoptera (orange tip labels) is not recovered as monophyletic for this orthogroup whereas the other orders are.

Figure S2. Alternative model structures for branch-site models that compare different taxonomic levels for assigning evolutionarily significant units (ESUs). In each tree, orders appear as Lepidoptera, Diptera, Coleoptera and Hymenoptera from top to bottom of the tree. Model structure -1 lumps together Diptera and Lepidoptera (the most derived orders) into a single unit. Black branches = within ESU branching, grey branches = among ESU branching. Structure -2 lumps Coleoptera, Diptera and Lepidoptera into a single unit. The remaining model structures all split one or more orders: for example, model CD splits both Coleoptera

and Diptera. Branch-site models were fitted to each model structure in turn and results compared using AIC to see which assignment of ESUs yields the best model fit.

Figure S3. Examples of simulations assuming a nearly neutral site model (model 1) and then analysed with either the same model (top row, A, B, C, D) or the nearly neutral branch-site model (model 3, E, F, G, H). The simulations either used the empirically reconstructed tree (A, E), or a tree with among-order branches artificially scaled to 1.5x (B, F), 2x (C, G) or 5x (D, H) their empirical value. The trees show branch lengths reconstructed by PAML with branch lengths in units of substitutions per codon (see scale bar) upon fitting the relevant model to the simulated dataset. The vertical side bars summarize the model estimates of the proportion of codons belonging to class 0 (light blue – uniformly under purifying selection), class 1 (white – uniformly neutral) or class 2a (red – shifting from purifying to neutral on among-order branches) from analysis of each simulated dataset.

Figure S4. **A)** An example gene tree for a single orthogroup. Black branches = within-order branches, grey = among-order branches. The scale bar is substitutions/site on the gene tree reconstructed by maximum likelihood in IQTree2. **B)** The corresponding amino acid alignment, with rainbow colours representing amino acids sorted by code alphabetically to indicate variability. The orthogroup is OG0000414 (includes ENSBMRT00000007056 transcript from the *Bibio* source GFF annotation file, which has a 89% identity, e-value = 7e-63 match to Synapse-associated protein of 47 kDa sequences using tblastx on NCBI).

Figure S5. Average GC% in the full concatenated alignment at first, second, third codon positions and across all position. Lepidoptera = green, Diptera = red, Coleoptera = orange, Hymenoptera = green. Separate bars are shown for the species sampled in each order. **A**) All codons. Note that 3rd position GC content varies greatly among species. 2nd position content is far less variable but greater variation is explained by ‘order’, as described in the main results text. **B**) Codons with >0.95 probability of belonging to class 2a when fitting model 4 (the positive selection branch-site model). Again, the strongest effect of ‘order’ is for 2nd positions rather than 3rd positions.

Figure S6. **A)** Mean amino acid composition across orders, sorted by whether the codon for the amino acid has a G or C at the 2nd position or not. ** indicates p<0.001 in anova of percentage composition versus order across species samples. **B)** Heatmap and clustering diagrams representing correlations in the changes across species among all 20 amino acids. A, R, G, P and W show the strongest patterns of significant variation among orders among those with G or C at 2nd position: they are cases with both 1st and 2nd position G or C, or for

W, amino acid is determined by 2nd and 3rd position both G. Note that R, H, I, L, K, M, F, T, W, and V are essential amino acids for insects, so among the GC amino acids with the strongest covariation with orders, R is essential, the others are non-essential.

Figure S7. Comparison of alternative models with different structures of evolutionarily significant units (ESUs) specified. Two example model structures are shown: model structure ‘D’ splits Diptera at its ancestral node (**top left**), ‘CD’ splits both Coleoptera and Diptera at their ancestral nodes (**top right**). ΔAIC (**middle barplot**) is the AIC score x10^4 of each model structure minus the AIC score of the best model, which is the model that splits Coleoptera and Diptera (CD). Split model structures are denoted by letters indicating the combination of one or more orders that are split in each model, ‘orders’ refers to our original model specifying each order as a single ESU. Model structures -1 and -2 lump orders together and both have high ΔAIC: -1 lumps Coleoptera, Diptera and Lepidoptera into a single ESU (**bottom left**), model -2 lumps Diptera and Lepidoptera (**bottom right**).

Figure S8. Multi-dimensional scaling plot of average pairwise amino acid divergence across the full concatenated alignment to show the clustering of each order. Each point represents a species, colour and letter coded by the order they belong to. Hymenoptera especially is very tightly clustered with respect to two-dimensional representation of amino acid variation, whereas Coleoptera especially is more widely dispersed.

FIGURE S1

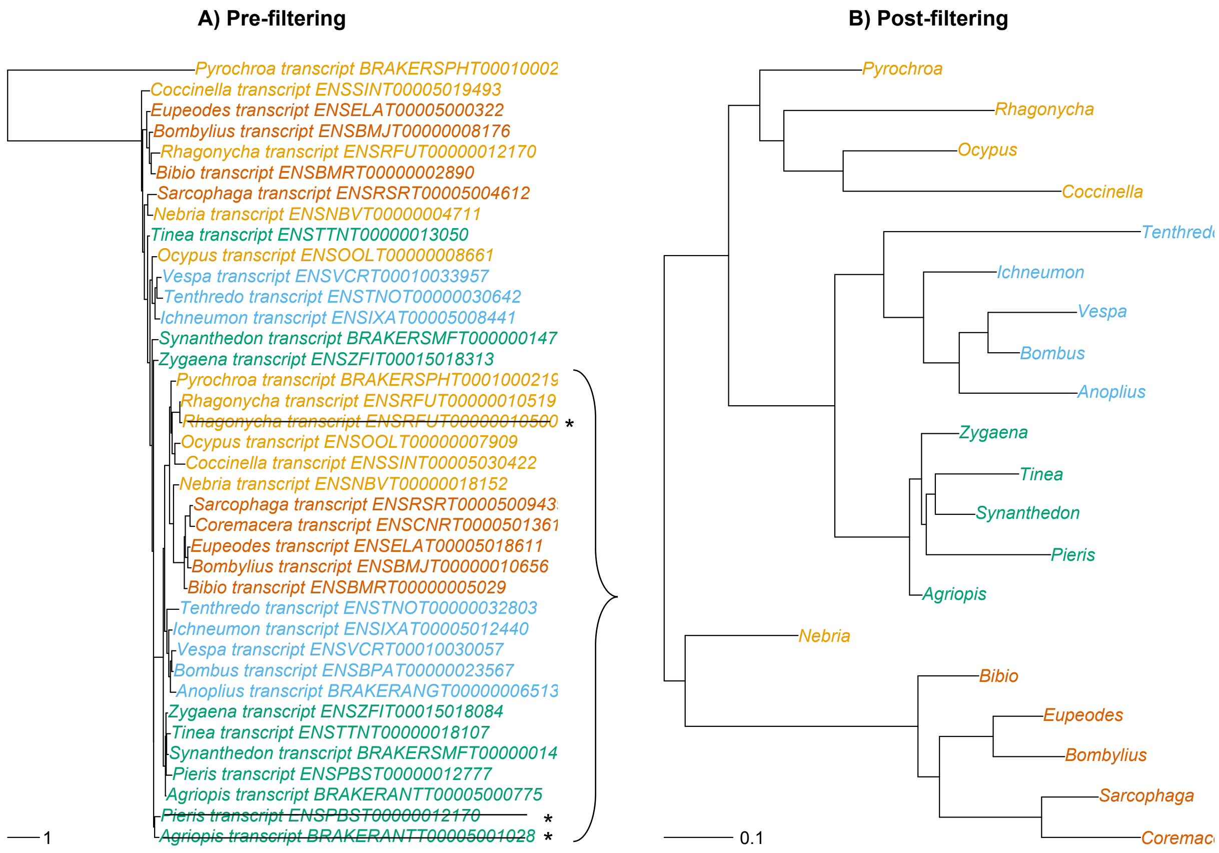

A) Pre-filtering
Pyrochroa transcript BRAKERSPHT00010002
Coccinella transcript ENSSINT00005019493
Eupeodes transcript ENSELAT00005000322
Bombylius transcript ENSBMJT00000008176
Rhagonycha transcript ENSRFUT00000012170
Bibio transcript ENSBMRT00000002890
Sarcophaga transcript ENSRSRT00005004612
Nebria transcript ENSNBVT00000004711
Tinea transcript ENSTTNT00000013050
Ocypus transcript ENSOOLT00000008661
Vespa transcript ENSVCRT00010033957
Tenthredo transcript ENSTNOT00000030642
Ichneumon transcript ENSIXAT00005008441
Synanthedon transcript BRAKERSMFT000000147
Zygaena transcript ENSZFIT00015018313
Pyrochroa transcript BRAKERSPHT0001000219
Rhagonycha transcript ENSRFUT00000010519
Rhagonycha transcript ENSRFUT00000010500 *
Ocypus transcript ENSOOLT00000007909
Coccinella transcript ENSSINT00005030422
Nebria transcript ENSNBVT00000018152
Sarcophaga transcript ENSRSRT0000500943
Coremacera transcript ENSCNRT0000501361
Eupeodes transcript ENSELAT00005018611
Bombylius transcript ENSBMJT00000010656
Bibio transcript ENSBMRT00000005029
Tenthredo transcript ENSTNOT00000032803
Ichneumon transcript ENSIXAT00005012440
Vespa transcript ENSVCRT00010030057
Bombus transcript ENSBPAT00000023567
Anoplius transcript BRAKERANGT00000006513
Zygaena transcript ENSZFIT00015018084
Tinea transcript ENSTTNT00000018107
Synanthedon transcript BRAKERSMFT00000014
Pieris transcript ENSPBST00000012777
Agriopis transcript BRAKERANTT00005000775
Pieris transcript ENSPBST00000012170 *
Agriopis transcript BRAKERANTT00005001028 *
1
B) Post-filtering
Pyrochroa
Rhagonycha
Ocypus
Coccinella
Tenthred
Ichneumon
Vespa
Bombus
Anoplius
Zygaena
Tinea
Synanthedon
Pieris
Agriopis
Nebria
Bibio
Eupeodes
Bombylius
Sarcophaga
Coremac
0.1

FIGURE S2

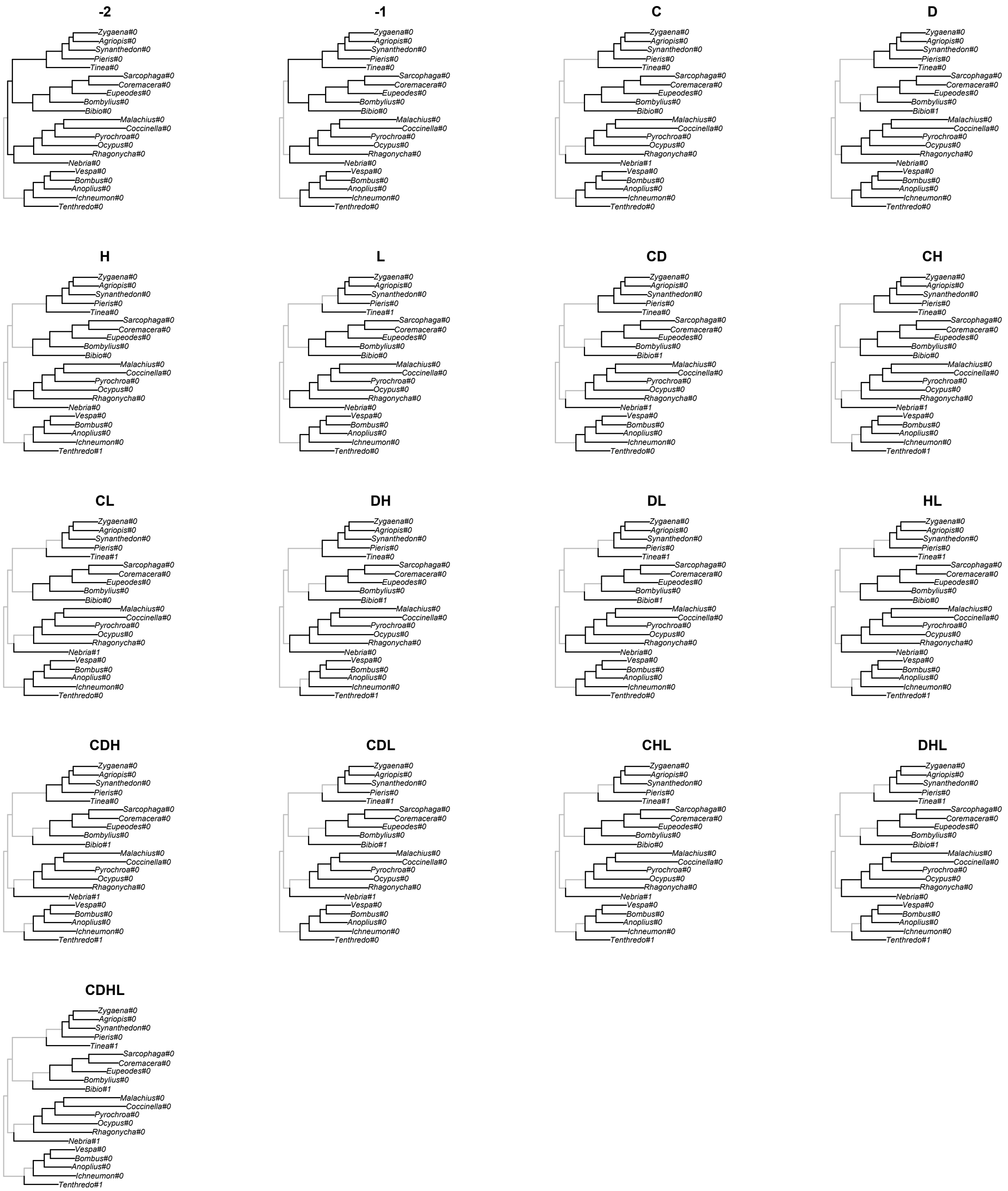
-2
Zygaena#0
Agriopis#0
Synanthedon#0
Pieris#0
Tinea#0
Sarcophaga#0
Coremacera#0
Eupeodes#0
Bombylius#0
Bibio#0
Malachius#0
Coccinella#0
Pyrochroa#0
Ocypus#0
Rhagonycha#0
Nebria#0
Vespa#0
Bombus#0
Anoplius#0
Ichneumon#0
Tenthredo#0
-1
Zygaena#0
Agriopis#0
Synanthedon#0
Pieris#0
Tinea#0
Sarcophaga#0
Coremacera#0
Eupeodes#0
Bombylius#0
Bibio#0
Malachius#0
Coccinella#0
Pyrochroa#0
Ocypus#0
Rhagonycha#0
Nebria#0
Vespa#0
Bombus#0
Anoplius#0
Ichneumon#0
Tenthredo#0
C
Zygaena#0
Agriopis#0
Synanthedon#0
Pieris#0
Tinea#0
Sarcophaga#0
Coremacera#0
Eupeodes#0
Bombylius#0
Bibio#0
Malachius#0
Coccinella#0
Pyrochroa#0
Ocypus#0
Rhagonycha#0
Nebria#1
Vespa#0
Bombus#0
Anoplius#0
Ichneumon#0
Tenthredo#0
D
Zygaena#0
Agriopis#0
Synanthedon#0
Pieris#0
Tinea#0
Sarcophaga#0
Coremacera#0
Eupeodes#0
Bombylius#0
Bibio#1
Malachius#0
Coccinella#0
Pyrochroa#0
Ocypus#0
Rhagonycha#0
Nebria#0
Vespa#0
Bombus#0
Anoplius#0
Ichneumon#0
Tenthredo#0
H
Zygaena#0
Agriopis#0
Synanthedon#0
Pieris#0
Tinea#0
Sarcophaga#0
Coremacera#0
Eupeodes#0
Bombylius#0
Bibio#0
Malachius#0
Coccinella#0
Pyrochroa#0
Ocypus#0
Rhagonycha#0
Nebria#0
Vespa#0
Bombus#0
Anoplius#0
Ichneumon#0
Tenthredo#1
L
Zygaena#0
Agriopis#0
Synanthedon#0
Pieris#0
Tinea#1
Sarcophaga#0
Coremacera#0
Eupeodes#0
Bombylius#0
Bibio#0
Malachius#0
Coccinella#0
Pyrochroa#0
Ocypus#0
Rhagonycha#0
Nebria#0
Vespa#0
Bombus#0
Anoplius#0
Ichneumon#0
Tenthredo#0
CD
Zygaena#0
Agriopis#0
Synanthedon#0
Pieris#0
Tinea#0
Sarcophaga#0
Coremacera#0
Eupeodes#0
Bombylius#0
Bibio#1
Malachius#0
Coccinella#0
Pyrochroa#0
Ocypus#0
Rhagonycha#0
Nebria#1
Vespa#0
Bombus#0
Anoplius#0
Ichneumon#0
Tenthredo#0
CH
Zygaena#0
Agriopis#0
Synanthedon#0
Pieris#0
Tinea#0
Sarcophaga#0
Coremacera#0
Eupeodes#0
Bombylius#0
Bibio#0
Malachius#0
Coccinella#0
Pyrochroa#0
Ocypus#0
Rhagonycha#0
Nebria#1
Vespa#0
Bombus#0
Anoplius#0
Ichneumon#0
Tenthredo#1
CL
Zygaena#0
Agriopis#0
Synanthedon#0
Pieris#0
Tinea#1
Sarcophaga#0
Coremacera#0
Eupeodes#0
Bombylius#0
Bibio#0
Malachius#0
Coccinella#0
Pyrochroa#0
Ocypus#0
Rhagonycha#0
Nebria#1
Vespa#0
Bombus#0
Anoplius#0
Ichneumon#0
Tenthredo#0
DH
Zygaena#0
Agriopis#0
Synanthedon#0
Pieris#0
Tinea#0
Sarcophaga#0
Coremacera#0
Eupeodes#0
Bombylius#0
Bibio#1
Malachius#0
Coccinella#0
Pyrochroa#0
Ocypus#0
Rhagonycha#0
Nebria#0
Vespa#0
Bombus#0
Anoplius#0
Ichneumon#0
Tenthredo#1
DL
Zygaena#0
Agriopis#0
Synanthedon#0
Pieris#0
Tinea#1
Sarcophaga#0
Coremacera#0
Eupeodes#0
Bombylius#0
Bibio#1
Malachius#0
Coccinella#0
Pyrochroa#0
Ocypus#0
Rhagonycha#0
Nebria#0
Vespa#0
Bombus#0
Anoplius#0
Ichneumon#0
Tenthredo#0
HL
Zygaena#0
Agriopis#0
Synanthedon#0
Pieris#0
Tinea#1
Sarcophaga#0
Coremacera#0
Eupeodes#0
Bombylius#0
Bibio#0
Malachius#0
Coccinella#0
Pyrochroa#0
Ocypus#0
Rhagonycha#0
Nebria#0
Vespa#0
Bombus#0
Anoplius#0
Ichneumon#0
Tenthredo#1
CDH
Zygaena#0
Agriopis#0
Synanthedon#0
Pieris#0
Tinea#0
Sarcophaga#0
Coremacera#0
Eupeodes#0
Bombylius#0
Bibio#1
Malachius#0
Coccinella#0
Pyrochroa#0
Ocypus#0
Rhagonycha#0
Nebria#1
Vespa#0
Bombus#0
Anoplius#0
Ichneumon#0
Tenthredo#1
CDL
Zygaena#0
Agriopis#0
Synanthedon#0
Pieris#0
Tinea#1
Sarcophaga#0
Coremacera#0
Eupeodes#0
Bombylius#0
Bibio#1
Malachius#0
Coccinella#0
Pyrochroa#0
Ocypus#0
Rhagonycha#0
Nebria#1
Vespa#0
Bombus#0
Anoplius#0
Ichneumon#0
Tenthredo#0
CHL
Zygaena#0
Agriopis#0
Synanthedon#0
Pieris#0
Tinea#1
Sarcophaga#0
Coremacera#0
Eupeodes#0
Bombylius#0
Bibio#0
Malachius#0
Coccinella#0
Pyrochroa#0
Ocypus#0
Rhagonycha#0
Nebria#1
Vespa#0
Bombus#0
Anoplius#0
Ichneumon#0
Tenthredo#1
DHL
Zygaena#0
Agriopis#0
Synanthedon#0
Pieris#0
Tinea#1
Sarcophaga#0
Coremacera#0
Eupeodes#0
Bombylius#0
Bibio#1
Malachius#0
Coccinella#0
Pyrochroa#0
Ocypus#0
Rhagonycha#0
Nebria#0
Vespa#0
Bombus#0
Anoplius#0
Ichneumon#0
Tenthredo#1
CDHL
Zygaena#0
Agriopis#0
Synanthedon#0
Pieris#0
Tinea#1
Sarcophaga#0
Coremacera#0
Eupeodes#0
Bombylius#0
Bibio#1
Malachius#0
Coccinella#0
Pyrochroa#0
Ocypus#0
Rhagonycha#0
Nebria#1
Vespa#0
Bombus#0
Anoplius#0
Ichneumon#0
Tenthredo#1

FIGURE S3

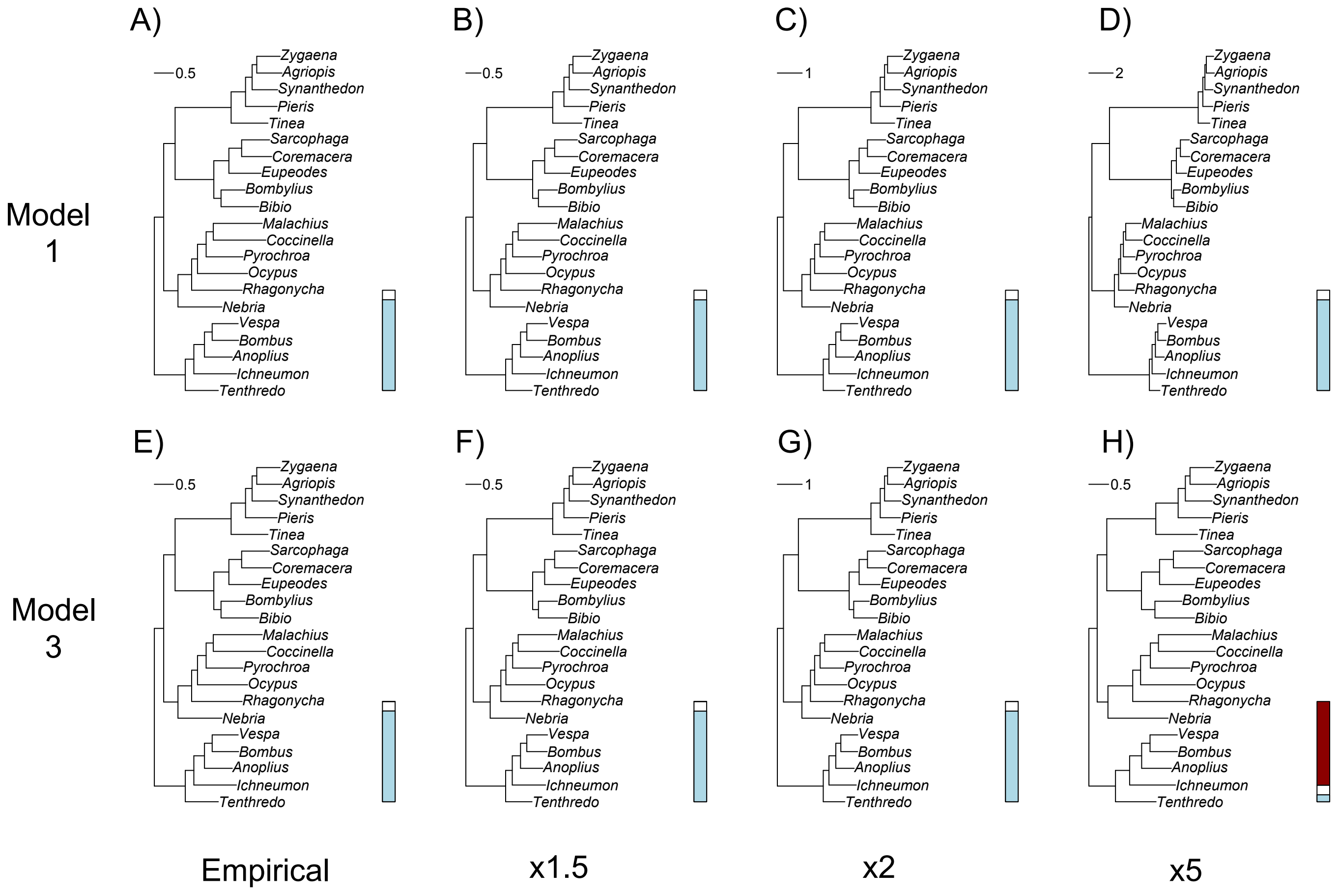
A)
B)
C)
D)
E)
F)
G)
H)
Model 1
Model 3
Empirical
x1.5
x2
x5
0.5
1
2
Zygaena
Agriopis
Synanthedon
Pieris
Tinea
Sarcophaga
Coremacera
Eupeodes
Bombylius
Bibio
Malachius
Coccinella
Pyrochroa
Ocypus
Rhagonycha
Nebria
Vespa
Bombus
Anoplius
Ichneumon
Tenthredo

FIGURE S4

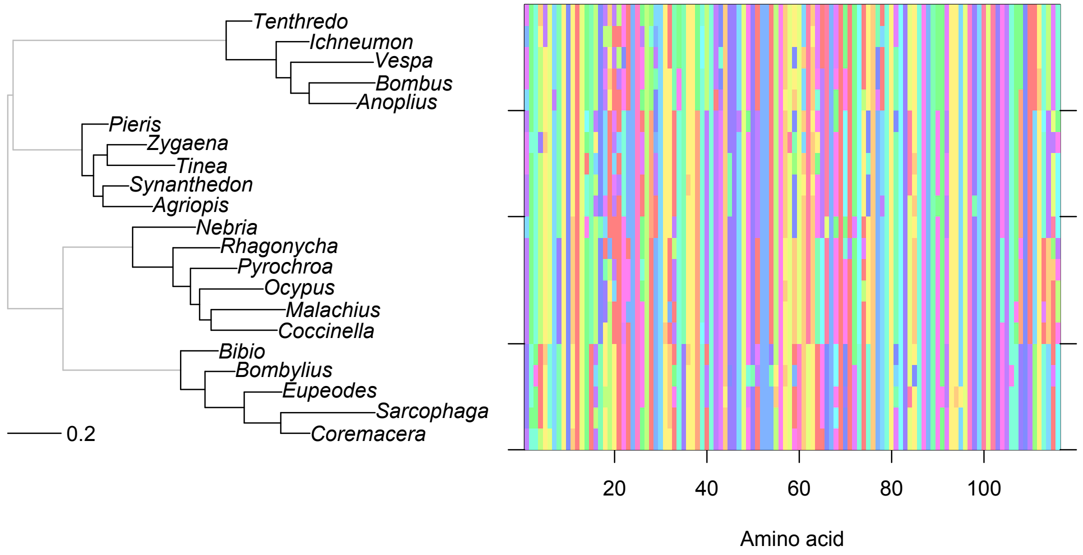

Tenthredo
Ichneumon
Vespa
Bombus
Anoplius
Pieris
Zygaena
Tinea
Synanthedon
Agriopis
Nebria
Rhagonycha
Pyrochroa
Ocypus
Malachius
Coccinella
Bibio
Bombylius
Eupeodes
Sarcophaga
Coremacera
0.2
20
40
60
80
100
Amino acid

FIGURE S5

A) All codons

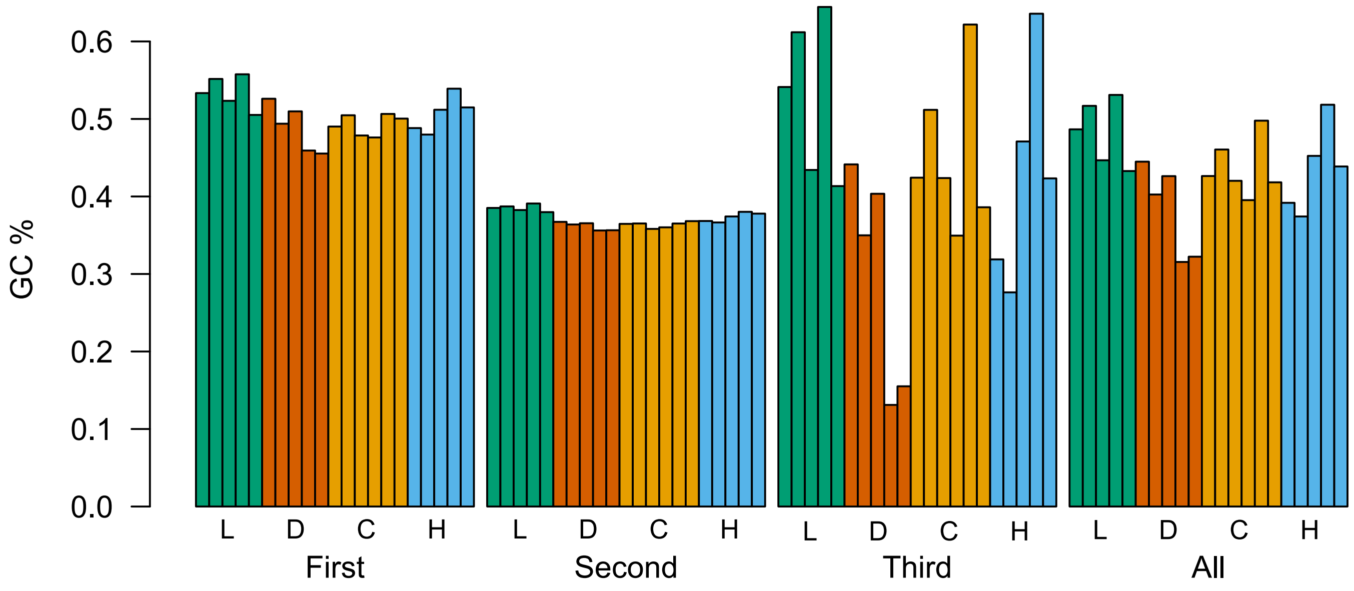

B) Codons with p>0.95 of belonging to class 2a

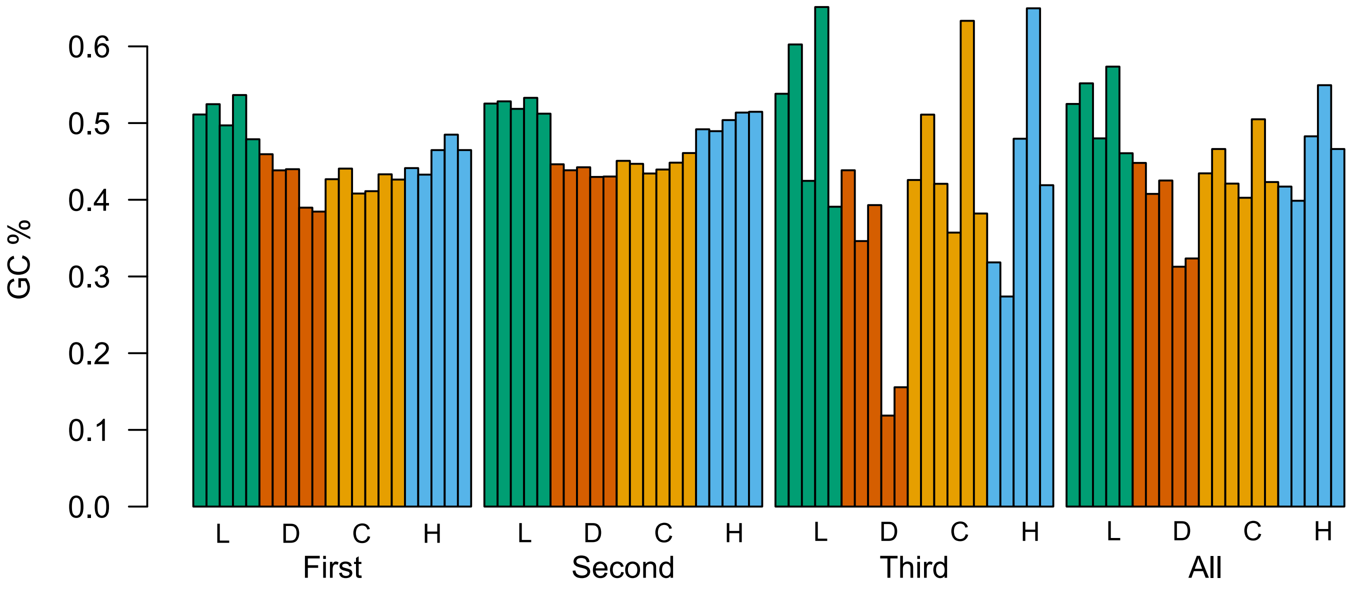

FIGURE S6

A)

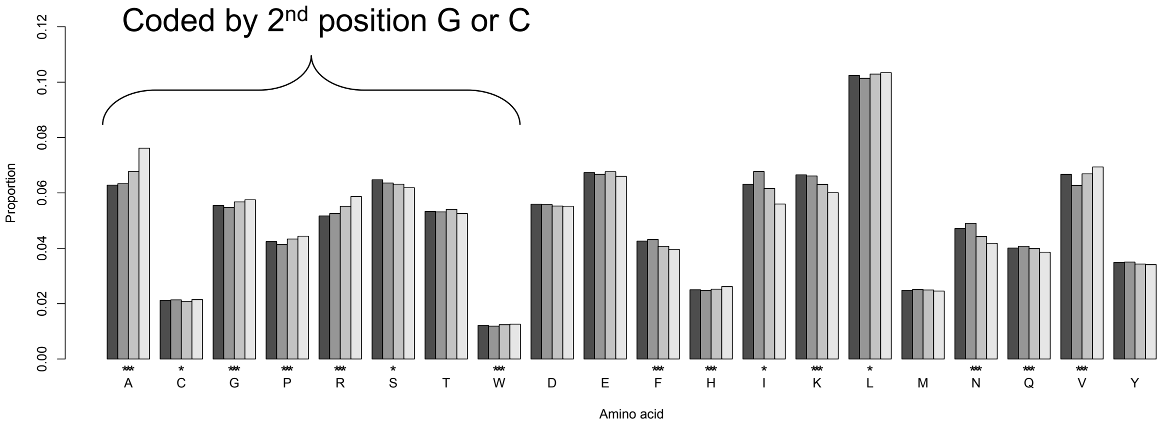

Coded by 2nd position G or C
Proportion
0.00
0.02
0.04
0.06
0.08
0.10
0.12
A
C
G
P
R
S
T
W
D
E
F
H
I
K
L
M
N
Q
V
Y
Amino acid

B)

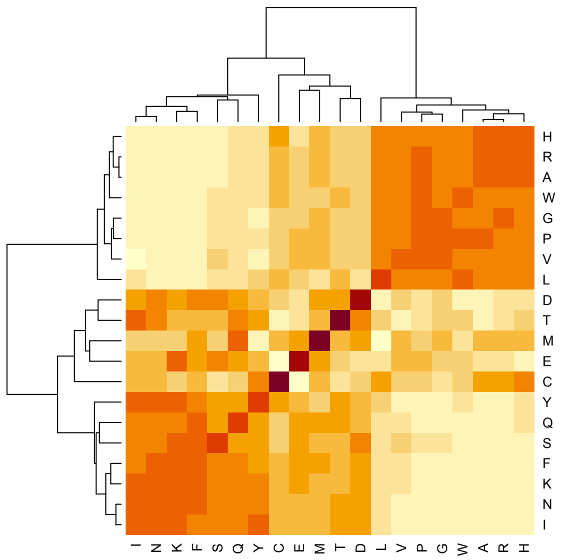

H
R
A
W
G
P
V
L
D
T
M
E
C
Y
Q
S
F
K
N
I
I N K F S Q Y C E M T D L V P G W A R H

FIGURE S7

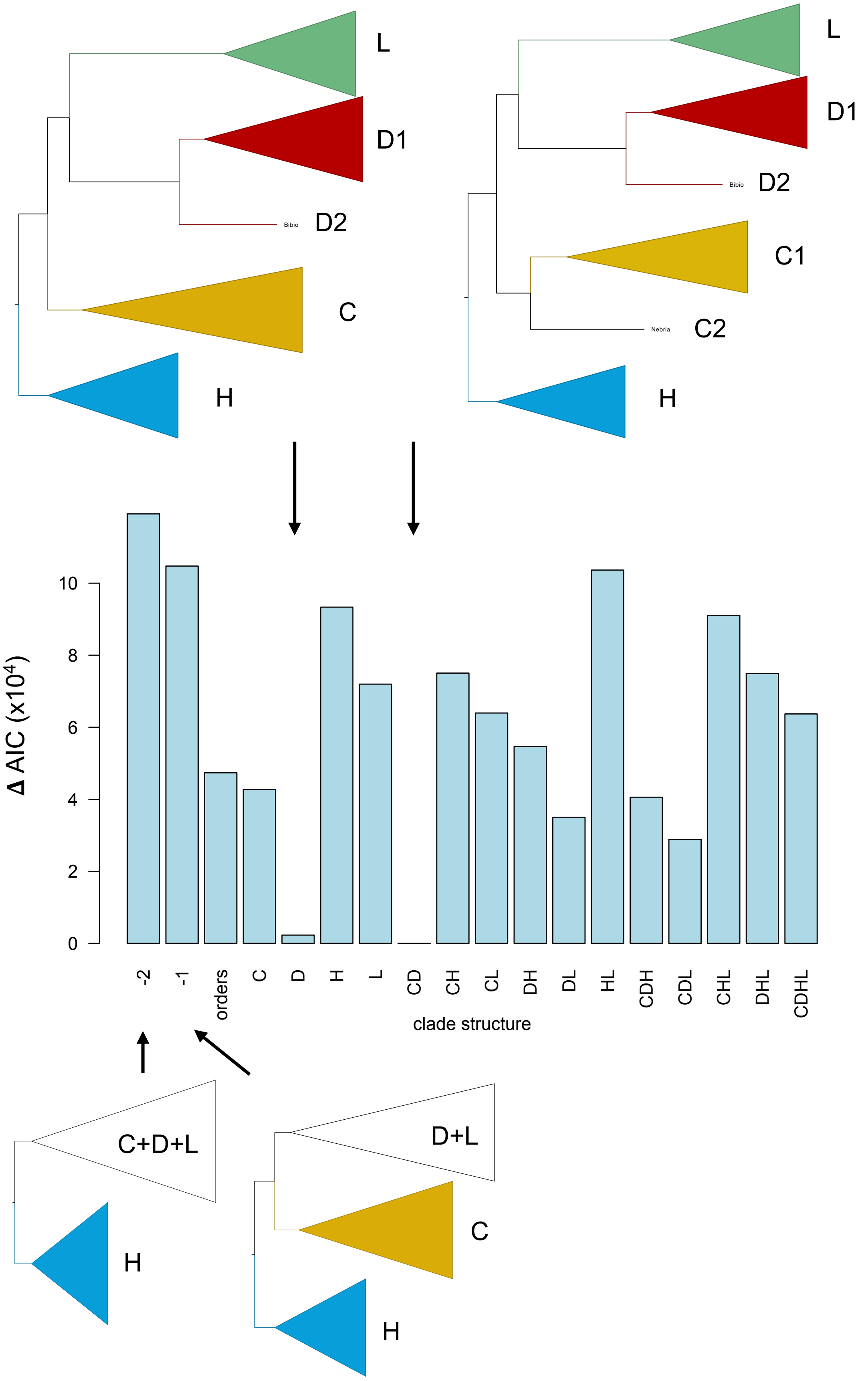
L
D1
Bibio
D2
C
H
L
D1
Bibio
D2
C1
Nebria
C2
H
Δ AIC (x10⁴)
10
8
6
4
2
0
-2
-1
orders
C
D
H
L
CD
CH
CL
DH
DL
HL
CDH
CDL
CHL
DHL
CDHL
clade structure
C+D+L
H
D+L
C
H

FIGURE S8

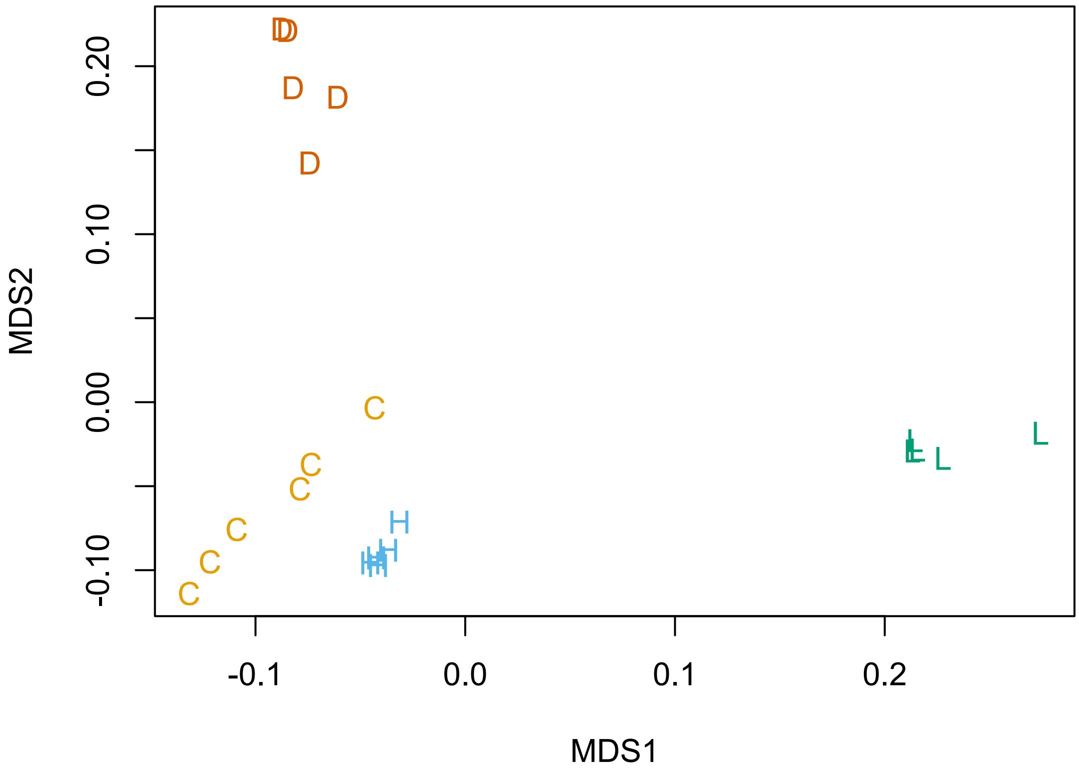

Supplement: qraf005_suppl_Supplementary_Figures_S1-S8 [file qraf005_suppl_supplementary_figures_s1-s8.pdf]
